# 2019 ISCB Innovator Award Recognizes William Stafford Noble

**DOI:** 10.1093/bioinformatics/btz396

**Published:** 2019-06-10

**Authors:** Christiana N Fogg, Ron Shamir, Diane E Kovats

**Affiliations:** Kensington, MD, USA; Computational Genomics Group, Blavatnik School of Computer Science, Tel Aviv University, Tel Aviv, Israel; International Society for Computational Biology, Leesburg, VA, USA

The ISCB Innovator Award honors an ISCB scientist who is within two decades of having completed his or her graduate degree and has made outstanding contributions to the field of computational biology. The 2019 winner is Dr. William Stafford Noble, Professor in the Department of Genome Science, University of Washington. Noble will receive his award and deliver a keynote presentation at the 2019 Joint International Conference on Intelligent Systems for Molecular Biology/European Conference on Computational Biology in Basel, Switzerland being held on July 21–25, 2019.

## William Stafford Noble—interested in learning stuff

**Figure:**
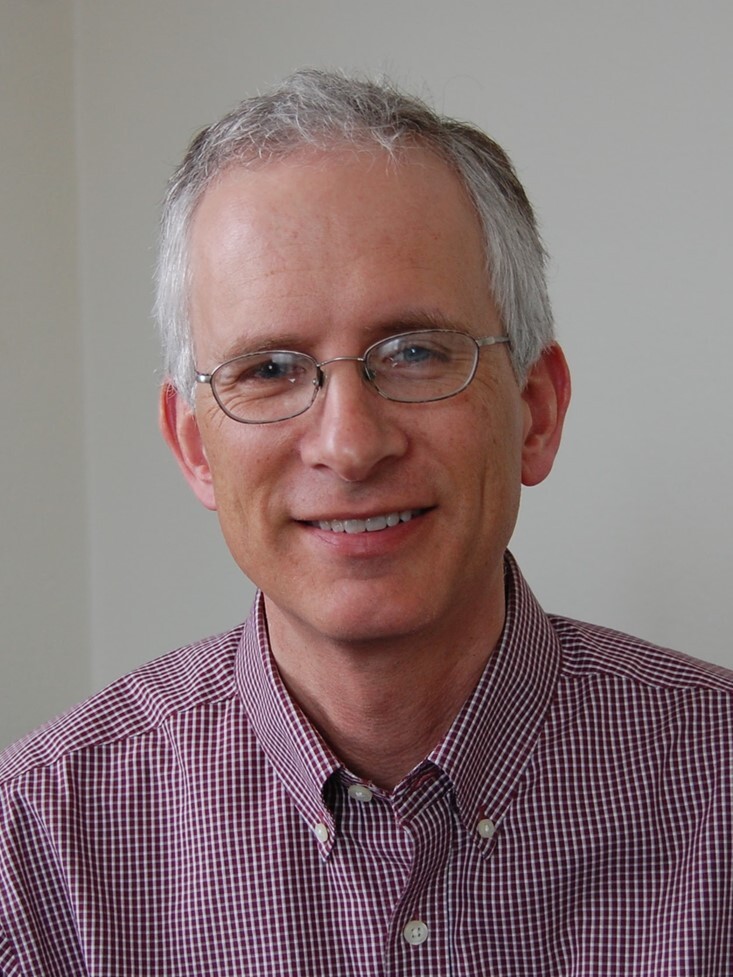


William Stafford Noble was raised in Naperville, IL, with his brothers and his parents who were both college professors. As a child, he didn’t have a specific interest in science, but he remembered, ‘I was just interested in learning stuff’. A simple test gave Noble a peek into his future career path. Noble recalled, ‘I took a career aptitude test in high school, and the results said I should be a college professor or computer scientist, but at that point I had never touched a computer’.

Noble went to Stanford University to complete a bachelor’s degree in Symbolic Systems, with a concentration in Philosophy. He has come to appreciate the multidisciplinary nature of his undergraduate degree, which included a broad range of coursework in computer science, cognitive science, linguistics, philosophy and mathematics. After graduating in 1991, Noble gained work experience in the field of speech recognition, and he also spent two years in the US Peace Corps in Lesotho, Africa. Noble said, ‘Both of my brothers went overseas after college, so I picked the Peace Corps. It seemed to be a little better organized than some other options’. Noble spent two years teaching math, physics and English literature to secondary students and had to develop teaching skills to explain complex material in a clear and straightforward way, training that has served him well throughout his career. All the while, he kept thinking about computer programming, and he would write down programs on paper in his free time. At the end of his first year in Lesotho, his parents visited him and brought him a laptop, so he could use the brief hours of evening electricity to transfer his programs from paper to a computer. Noble also developed an interest at this time in artificial life, which was a relatively new field. He got his hands on several artificial life conference proceedings and set off to study this area as a newly minted graduated student at the University of California, San Diego in 1994. Relatively quickly, he came to feel that this field was too descriptive, so he began to search for a different dissertation subject. His future Ph.D. mentor, Charles Elkan, emailed him about a funding opportunity that would allow him to study hidden Markov models (HMMs) in protein and DNA sequences. Noble was open to this topic because he was already familiar with HMMs from his work in speech recognition, and he went on to complete his Ph.D. in computer science and cognitive science in 1998. Noble’s first bioinformatics publication, which was based on his Ph.D. research, described a web server for motif-based sequence analysis (the MEME Suite) that is still in use today.

Noble went on to David Haussler’s lab at the University of California, Santa Cruz as a Sloan/DOE postdoctoral fellow and co-authored the first paper that applied support vector machines to microarray gene expression data. He also developed kernel functions that could be used to represent a variety of data types, and he showed how kernels could be used to perform inference jointly from these heterogenous types of data. This work was ultimately developed into applications in inference of protein-protein interactions and gene function that are used by many researchers.

In 1999, Noble became an Assistant Professor in the Department of Computer Science at Columbia University, with a joint appointment at the Columbia Genome Center. He moved to his current appointment at the University of Washington in 2002 in the newly formed Department of Genome Sciences with adjunct appointments in the Department of Computer Science and Engineering, the Department of Medicine and the Department of Biomedical Informatics and Medical Education. As an independent investigator, Noble has expanded his research interests including the development of unsupervised machine learning methods for semi-automated genome annotation, and the application of machine learning and statistical methods to analyze proteomic data. He has also worked with collaborators to develop high-throughput assays to characterize the 3D structure of DNA in the nucleus.

Throughout his career, Noble has grown as a scientist and mentor by learning from those who have mentored him, as well as observing how his collaborators mentor students and run their labs. Noble also credits his wife, Nancy Stafford Noble, for being a valuable sounding board and providing her expertise as an executive coach as he has navigated the many challenges of being a PI. Noble’s prodigious body of work includes authorship of over 230 peer-reviewed articles. He has trained and advised 15 graduate students and 21 postdoctoral fellows, many of whom now hold faculty appointments, and he was honored with the Postdoc Mentor of the Year Award by the University of Washington Postdoctoral Association.

Outside of the lab, Noble is an active member of the global computational biology community through his service on multiple editorial boards, conference committees, study sections and roles on the ISCB Board and various committees. Noble has been a part of ISCB since its early years and has always felt at home at ISMB meetings, which he considers one of the few gatherings that brings together computational biologists who bridge the gap between basic computer science and applications in biology. Noble feels deeply honored by his recognition with the 2019 ISCB Innovator Award, particularly as this award is bestowed upon him by colleagues for whom he holds great respect and admiration.

